# Differentiation measures for conservation genetics

**DOI:** 10.1111/eva.12590

**Published:** 2018-01-29

**Authors:** Lou Jost, Frederick Archer, Sarah Flanagan, Oscar Gaggiotti, Sean Hoban, Emily Latch

**Affiliations:** ^1^ Fundacion EcoMinga Baños Ecuador; ^2^ Southwest Fisheries Science Center La Jolla CA USA; ^3^ National Institute for Mathematical and Biological Synthesis University of Tennessee Knoxville TN USA; ^4^ School of Biology Scottish Oceans Institute University of St Andrews St Andrews UK; ^5^ The Morton Arboretum Lisle IL USA; ^6^ Department of Biological Sciences University of Wisconsin‐Milwaukee Milwaukee WI USA

**Keywords:** fixation indices, genetic differentiation indices, genetic diversity

## Abstract

We compare the two main classes of measures of population structure in genetics: (i) fixation measures such as *F*_ST_,*G*_ST_, and θ and (ii) allelic differentiation measures such as Jost's *D* and entropy differentiation. These two groups of measures quantify complementary aspects of population structure, which have no necessary relationship with each other. We focus especially on empirical aspects of population structure relevant to conservation analyses. At the empirical level, the first set of measures quantify nearness to fixation, while the second set of measures quantify relative degree of allelic differentiation. The two sets of measures do not compete with each other. Fixation measures are often misinterpreted as measures of allelic differentiation in conservation applications; we give examples and theoretical explanations showing why this interpretation can mislead. This misinterpretation has led to the mistaken belief that the absolute number of migrants determines allelic differentiation between demes when mutation rate is low; we show that in the finite island model, the absolute number of migrants determines nearness to fixation, not allelic differentiation. We show that a different quantity, the factor that controls Jost's *D*, is a good predictor of the evolution of the actual genetic divergence between demes at equilibrium in this model. We also show that when conservation decisions require judgments about differences in genetic composition between demes, allelic differentiation measures should be used instead of fixation measures. Allelic differentiation of fast‐mutating markers can be used to rank pairs or sets of demes according to their differentiation, but the allelic differentiation at coding loci of interest should be directly measured in order to judge its actual magnitude at these loci.

## 1 INTRODUCTION

Populations of threatened species are often subdivided into demes that are isolated from each other to some degree. Conservation biologists and resource managers need to know the structure of these populations in order to implement effective management policies, for example, to prioritize protection status or to determine translocation options. The geneticist's toolbox contains two broad families of measures that quantify population structure—fixation measures like *F*_ST_, *G*_ST_, and θ, and allelic differentiation measures, like Jost's *D* and entropy differentiation.

In the literature, there has been much discussion of the purpose, application, and performance of these two families of metrics (Gerlach, Jueterbock, Kraemer, Deppermann, & Harmand, 2010; Heller & Siegismund, 2009; Jost, 2008; Meirmans & Hedrick, 2011; Whitlock, 2011). Confusion often arises due to ambiguity in the term “differentiation.” Sometimes the two families have been treated as rivals, or as if members of the second family are “correcting” or estimating the members of the first. In reality, the two families are designed to quantify complementary aspects of population structure. Thus, attempts to compare them as if they are measuring the same feature are improper. The question under investigation should determine which family is used. If the wrong family of measures is used for a given application, invalid inferences will be drawn, which could put species at risk if they serve as the basis for management decisions. This article explains how and why these two families of measures differ, contrasts their behavior, points out some common misconceptions about *G*_ST_, and shows how to choose the appropriate measure for a given application. For didactic purposes, we concentrate on the simplest heterozygosity‐based representatives of each family, *G*_ST_ and *D*. Both sets of measures are zero when there is no structure (except that *G*_ST_ is undefined when all demes are fixed for the same allele), but apart from this case, they differ in their relative sensitivity to fixation and differentiation.

## 2 THE TWO MAIN FAMILIES OF MEASURES

The oldest and most popular measure of population structure, *F*_ST_, was developed by Wright (1943, 1965). It measures “the probability that two homologous genes, chosen at random from the subpopulation, are both descended from a gene in the subpopulation” (Crow & Kimura, 1970). This quantity depends only on pedigrees, not on the actual differentiation between the alleles. Nei (1973) later introduced *G*_ST_, a multi‐allele generalization of *F*_ST_, which was defined as the relative difference between the expected heterozygosity of the whole population *H*_*T*_ and the mean expected heterozygosity of the individual demes *H*_*S*_:

(1) $$G_{\mathrm{ST}}=\left(H_{T}-H_{S}\right)/H_{T}=1-H_{S}/H_{T},$$

As *G*_ST_ is a function only of the allele frequencies, it is not measuring exactly the same thing as the original concept of *F*_ST_. Weir and Cockerham (1984) introduced θ as an unbiased estimator of *F*_ST_. Collectively, these three measures and their relatives share many properties and make up the first family of measures of population structure. This family measures a kind of demographic differentiation. We refer to them as “fixation” measures, because at the empirical level, they mainly reflect nearness to fixation in each deme rather than the actual degree of differentiation of allele frequencies between the demes. Wright (1978) recognized this distinction, which we shall use throughout this paper. He explained that his fixation index *F*_ST_ “…is thus not a measure of the degree of differentiation in the sense implied in the extreme case by the absence of any common allele. It measures differentiation within the total array in the sense of the extent to which the process of fixation has gone toward completion… In using the latter [*F*_ST_], it must again be borne in mind that it measures the degree of completion of the process of fixation, not absolute differentiation” (p 84, Wright, 1978). Wright did not seek a measure of allelic differentiation, because that kind of differentiation necessarily varies among loci according to their mutation rates. Wright was seeking a measure that was sensitive only to demographic variables (namely population size and migration rate), which affect all loci equally. In this article, we will use *G*_ST_ to represent this family because of its analytic simplicity, but the other members of this family (*F*_ST_ and θ) have broadly similar behaviors.

Fixation measures are commonly used not only to describe the nearness to fixation of a set of demes, but also to infer the values of the demographic parameters that cause the observed pattern. The factors that control the value of *G*_ST_ can be identified by studying simple model systems such as Wright's finite island model. In that model, with infinite alleles, the expected equilibrium value of *G*_ST_ can be expressed analytically as a function of the model parameters *N* (number of individuals per deme), *d* (number of demes), *m* (migration rate per generation), and μ (mutation rate per generation):

(2) $$G_{\mathrm{ST}}\approx 1/\left[{\left(d/\left(d-1\right)\right)}^{2}4\mathrm{Nm}+\left(d/\left(d-1\right)\right)4N\mu +1\right].$$

**Figure 1:**
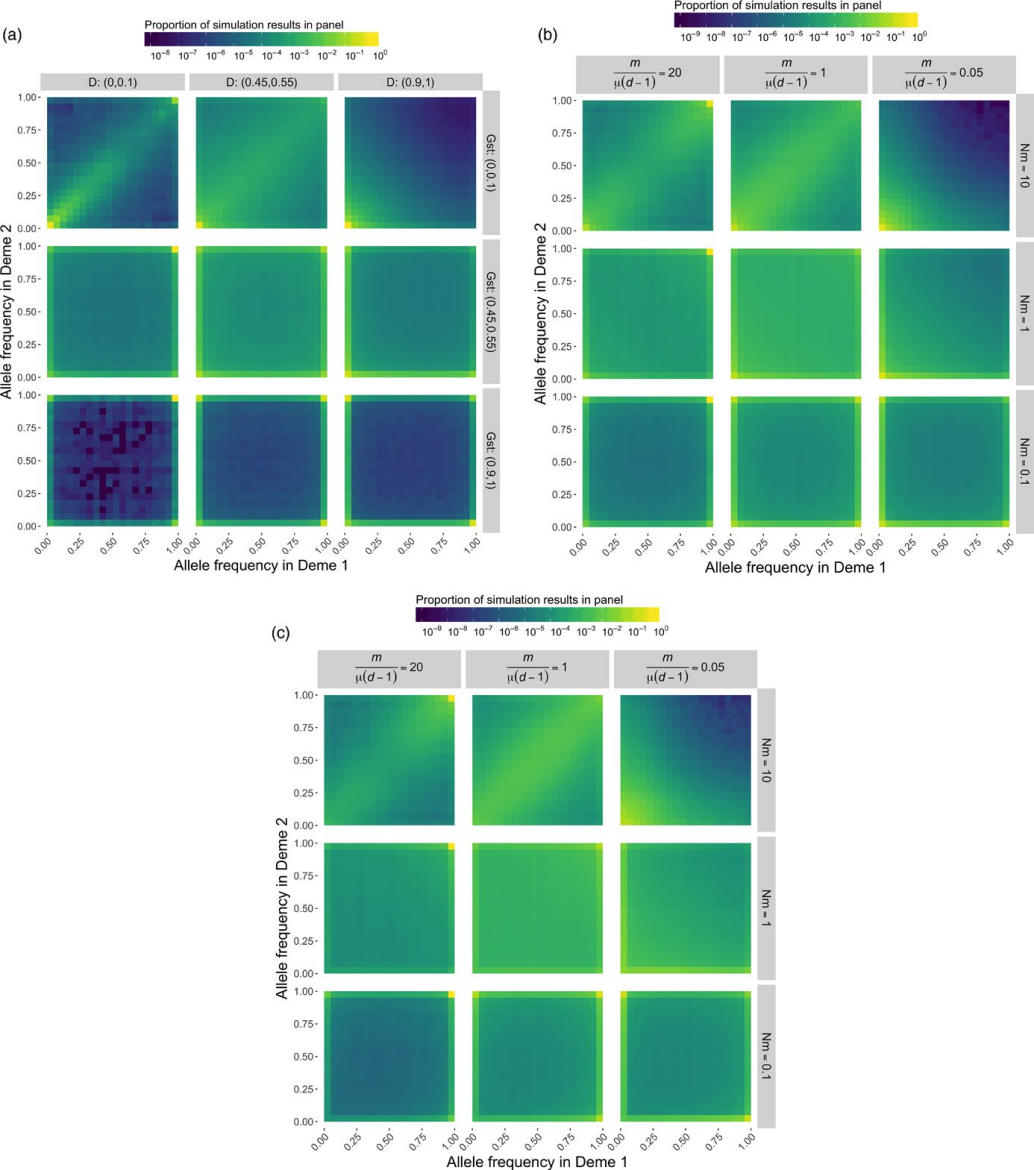
Heatmaps presenting the joint allele frequency spectrum under various evolutionary scenarios. We run 1,000 replicates for each evolutionary scenario consisting of different combinations of (a) *D* and *G*_ST_, and (b,c) *m*/μ(*d*−1) and *Nm* values for a single locus evolving under the infinite‐allele model. In a and b, the color represents the proportion of simulation results for which the frequency of distinct alleles is x in deme 1 and y in deme 2. In c, the points from b have been weighted by the mean allele frequency to downweight rare alleles. Note that as opposed to a typical joint allele frequency plot that presents the derived allele frequency for bi‐allelic loci, here we consider the frequencies of all distinct alleles at a locus. Furthermore, the plots comprise all pairwise combinations of demes. When allelic differentiation is low, most of the simulation runs will yield points that lie close to the line *x* = *y*, indicating allele frequencies are about the same in both demes. When allelic differentiation is high, allele frequencies are concentrated along the *x*‐ and *y*‐axes, indicating allele frequencies are near zero in one of the two demes. Fixation is indicated when the points occupy the upper or rightmost edges of the panels (the lines *x* = 1 or *y* = 1). The plots show that high values of *G*_ST_ indicate a high degree of fixation. A full description of the demographic parameters used and simulation methods are given in Supporting Information

This quantity controls the expected nearness to fixation. When *G*_ST_ is high, demes tend to be fixed for a single allele (Figure 1a, bottom row). When *m* ≫ μ, this formula shows that *G*_ST_ depends only on the absolute number of migrants *Nm* and the number of demes *d*. Therefore in this case the expected value of *G*_ST_ is the same for all loci whose mutation rates are much smaller than the migration rate. Estimates of *G*_ST_ from many such loci can thus be averaged and used to estimate the absolute number of migrants, provided all other assumptions of the island model are met. The absolute number of migrants per generation is the factor that controls nearness to fixation at such loci in a set of demes.

However, in conservation genetics applications and in many theoretical evolutionary applications (such as the study of speciation), the interest of the investigator is more often the actual amount of allelic differentiation between demes, especially at loci that affect the viability of the species under study. A second family of metrics addresses Wright's “absolute differentiation,” which is differentiation “in the sense implied in the extreme case by the absence of any common allele.” These metrics equal unity when each deme consists entirely of private alleles, and equal zero when all demes are identical (having the same alleles at the same frequencies). We call this aspect of population structure “allelic differentiation.” The family of allelic differentiation measures includes Jost's *D*, a heterozygosity‐based measure which was introduced to genetics from ecology to describe this aspect of population structure:

(3) $$\begin{array}{cc} D= & \left[\left(H_{T}-H_{S}\right)/\left(1-H_{S}\right)\right]*\left[d/\left(d-1\right)\right]=1-J_{\mathrm{between}}/J_{\mathrm{within}}=\\ & 1-\exp(-\text{NGD}), \end{array}$$

where *d* is the number of demes, *J* is Nei's gene identity (Nei, 1973), and NGD is Nei's genetic distance (Nei, 1972). It was derived from a mathematical analysis of the relation between the abstract concepts of diversity and differentiation (Jost, 2007, 2008). Note that *D* is not an estimator of *G*_ST_, despite often frequently being used as such (e.g., Wang, 2012). Members of this second family differ from each other in their weighting of allele frequencies. Jost's *D* is the member of this family which weighs alleles by the square of their frequencies, the same weighting used by heterozygosity.

Under the finite island model with infinite alleles, the expected equilibrium value of the allelic differentiation measure *D* is:

(4) D≈1/{1+m/[μ(d−1)]}.

Allelic differentiation between demes is controlled by *m*/[μ(*d*−1)], not *Nm* (Figure 1b,c). This is the same quantity that controls Nei's genetic distance between demes, and *D* is a simple monotonic function of that genetic distance (Jost, 2009). Unlike nearness to fixation, which is nearly independent of μ if μ ≪ *m*, the allelic differentiation between demes generally varies from locus to locus, because its equilibrium value always depends strongly on the mutation rate. This is a real effect, not a flaw in the differentiation measures (Figure 1). Thus, if the absolute magnitude of the allelic differentiation at a locus is important, *D* should be estimated from the loci we care about, or other loci expected to have similar mutation rates, and not from fast‐mutating markers. However, measurements of *D* from fast‐mutating markers are still useful for ranking pairs of demes according to their degree of allelic differentiation across the genome.

Equation (4) is also valid for the harmonic means of *D*_*i*_ and μ_*i*_ across *i* loci. For sets of loci whose per‐base mutation rate μ_*b*_ is approximately constant, the harmonic mean of *D* across loci is approximately (for large loci):

(5) $$\tilde{D}\approx 1/\left\{1+m/\left[\left(d-1\right)\tilde{L}\mu_{b}\right]\right\}.$$

where *L* is the harmonic mean of the lengths (i.e., the number of basepairs) of each locus.

The same general formula for partitioning diversity, and for normalizing the between‐group component of diversity to generate a measure of allelic differentiation, can be applied to other diversity measures besides those based on heterozygosity. This generates a parametric family of differentiation measures that vary in the weight q given to allele frequencies. Jost's *D* is obtained when *q* = 2. The general formula is

(6) $${}^{q}\text{Differentiation}=1-\left[{{(}^{q}D_{S}{/}^{q}D_{T})}^{q-1}-{\left(1/d\right)}^{q-1}\right]/\left[1-{\left(1/d\right)}^{q-1}\right],$$

(the one‐complement of the similarity measure equation 6.12 in Jost, Chao, & Chazdon, 2011) where ^*q*^ *D*_*T*_ and ^*q*^ *D*_*S*_ are the total and mean within‐deme diversities of order *q* based on Hill numbers (Jost, 2007; Gaggiotti et al., 2018). Note that for *q* ≠ 0 or 1, the statistical weights of each deme must be taken to be equal if the goal is to compare differentiation of the relative frequencies of the alleles between demes. Note also that the quantity ^*q*^ *D*_*T*_/^*q*^ *D*_*S*_ is the between‐group diversity (Hill number) of order *q*, so these differentiation measures are all simply normalizations of that between‐group diversity for different values of *q*.

Besides *D*, two other members of this family are especially useful. The allelic differentiation measure based on Shannon entropy, which we here write as *E*_ST,_ weighs alleles by their population frequency. It is obtained by taking the limit of Equation (6) as *q* approaches unity (Jost, 2007; Sherwin, 2010):

(7) $$E_{\mathrm{ST}}=\left(E_{T}-E_{S}\right)/E_{w},$$

where *E*_*T*_ and *E*_*S*_ are the total Shannon entropy of the pooled demes and the mean within‐deme Shannon entropy (weighted by deme size), respectively, and *E*_*w*_ is the entropy of the relative sizes of each deme. This measure is especially useful when relative sizes of the demes differ. In contrast, the heterozygosity‐based differentiation measure *D* assigns all demes equal statistical weights (this option is also available for *E*_ST_ if the weights are not known or if they are irrelevant to the question under investigation). The entropy‐based measure obeys stronger monotonicity properties than *D* (Jost et al., 2010; Gaggiotti et al., 2018). *D* weighs alleles according to the square of their relative frequencies (as it is based on heterozygosity), so it mainly measures the differentiation of the most common alleles. Therefore, when some of the most common alleles are shared and some are not, sometimes adding a new low‐frequency private allele to a deme can slightly reduce *D*, because adding this allele reduced the squared relative frequency of more common unshared alleles in the deme. *E*_ST_ on the other hand always increases with the addition of a private allele, a feature that is sensible in most conservation applications. Its expected value under the finite island model was recently derived (Chao et al., 2015).

The allelic differentiation measure based on allele number (the one‐complement of the multiple‐community Sorensen index of ecology derived in Jost, 2007) derived from Equation (6) when *q* = 0, is

(8) $$K_{\mathrm{ST}}=1-\left[\left(K_{T}/K_{S}-d\right)/\left(1-d\right)\right],$$

where *d* is the number of demes, *K*_*T*_ is the total number of alleles, and *K*_*S*_ is the (unweighted) mean of the number of alleles per deme. *K*_ST_ gives the differentiation of the demes in terms of the relative number of shared alleles among the demes, without regard to their frequencies. This measure possesses the strongest monotonicity properties of all differentiation measures, so it provides useful information for conservation managers. Caballero and Garcia‐Dorado (2013) and Vilas, Pérez‐Figueroa, Quesada, and Caballero (2015) demonstrate some of the advantages of diversity and differentiation measures based on allele numbers.

The kind of population structure measured by *D*, and other members of the allelic differentiation family of measures, is complementary to the kind of population structure measured by fixation measures such as *G*_ST_. One family can be close to zero while the other family can be close to unity. This is not an estimation problem (e.g., insufficient sample size, biased sampling); these differences arise even for the true population values of the measures. In this article, we will always be discussing the true population values of these measures, unless otherwise specified. The next section shows how the families differ at the descriptive, empirical level.

## 3 WHAT EMPIRICAL ASPECTS OF POPULATION STRUCTURE DO THESE TWO FAMILIES QUANTIFY?

At a descriptive or empirical level, we are not making any inferences about underlying processes; we only seek to describe aspects of the actual allele distribution at the present moment. This will often be the most appropriate level for conservation analyses. In focusing on the actual magnitudes of *D* and *G*_ST_, we directly measure the aspects of population structure that matter to us, rather than make dubious inferences based on unrealistic and unverifiable assumptions about equilibrium. We must emphasize that neither *G*_ST_ nor *D* should be used to estimate or make statements about current migration, although this was unfortunately common practice in conservation genetics until recently (see Whitlock & McCauley, 1999 and references therein for a detailed review). The current values of *G*_ST_ and *D* will be a consequence of an accumulation of historic and recent migration and population sizes; populations that are currently completely isolated from migration can still show low *G*_ST_ and high *m* using this formula. Because *D* is independent of within‐group diversity, it is more stable with respect to variations in deme size, but past variation in migration rate can still leave its mark. Populations of threatened species are by definition not at equilibrium; we are concerned about them precisely because their deme numbers and/or deme sizes and migration rates have experienced important recent reductions. For example, bottlenecks and fragmentation of habitat can lead to smaller population sizes, reduced genetic diversity, and reduced gene flow among populations. These events do not have to be ecologically recent to have an effect on fixation and differentiation statistics (Leng & Zhang, 2013).

At this empirical level, the fixation index *G*_ST_ and the allelic differentiation measure *D* behave more or less as their respective names suggest. Their differences are best illustrated by examining the kinds of population structure that minimize and maximize their values.

### 3.1 Infinite‐allele case

Let us assume the infinite‐allele model, so that there is no strict upper limit to the possible number of alleles at a locus. In practice, this is a valid approximation for loci comprised of many base pairs. The fixation measure *G*_ST_ will take its maximum value of unity when all demes are fixed for a single allele. It does not matter whether different alleles are fixed in each deme, or the same allele is fixed in nearly all the demes; in both of these cases, we will obtain exactly *G*_ST_ = 1.00 at the locus under study because *fixation* has occurred. On the other hand, allelic differentiation measures like *D* will take their maximum value of unity if and only if the demes share no alleles. The kinds of population structure that maximize *G*_ST_ are not the same as those that maximize *D*. When *G*_ST_ is unity, *D* can be either large or small, and vice versa (Figure 1a).

**Figure 2:**
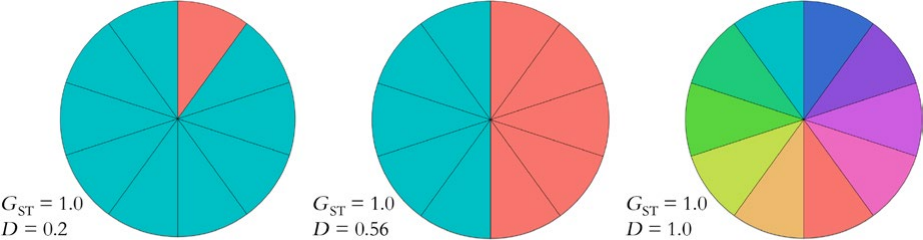
Three scenarios with ten demes that have gone to fixation. Each slice represents a deme, and its color indicates the identity of its fixed allele. Each unique allele is represented by a distinct color. *G*_ST_ in each scenario is 1.0, but *D* ranges from 0.2 when almost all demes are fixed for the same allele (left) to 1.0 when all demes are fixed for different alleles (right)

Imagine a scenario with three species, each of which has ten demes that have gone to fixation (Figure 2). *G*_ST_ could equal 1 when nine of the ten demes share the same allele that has been fixed, or when half of the demes share one allele and the other half share another allele, or when all ten demes are fixed for different alleles. If a conservation manager had only used global *G*_ST_ to decide how many demes to protect in each of these three species, he or she would have sought to protect all demes in each case. In fact at this locus, the diversity of the first species would be conserved by saving just two demes, while the diversity of the third species requires conserving all ten demes. These differences in diversity are captured by the three different values of *D* (0.2, 0.56, and 1.0, respectively).

We can gain more insight into the difference between these measures by examining the first species of Figure 2. The ten demes have all gone to fixation at the locus of interest, and nine of the ten demes are genetically identical, that is, fixed for the same allele. If we wanted to describe the degree of allelic differentiation among this set of demes based on first principles, we might start by writing down all 45 (10*9/2) possible pairwise comparisons between the ten demes. For each pair of demes in this list, if they are fixed for the same allele, their allelic differentiation would be zero, and if they are fixed for different alleles, their allelic differentiation would be unity. In this case, nine of the pairwise comparisons are between completely differentiated demes, and 36 of the pairwise comparisons are between genetically identical demes. The mean pairwise differentiation is thus (9*1 + 36*0)/45 = 0.20. This is exactly the value of *D* for this set of demes. *G*_ST_ on the other hand is equal to unity, even though almost all demes are fixed for the same allele. This is the correct value for a measure of nearness to fixation, but it shows that nearness to fixation has little to do with allelic differentiation.

**Figure 3:**
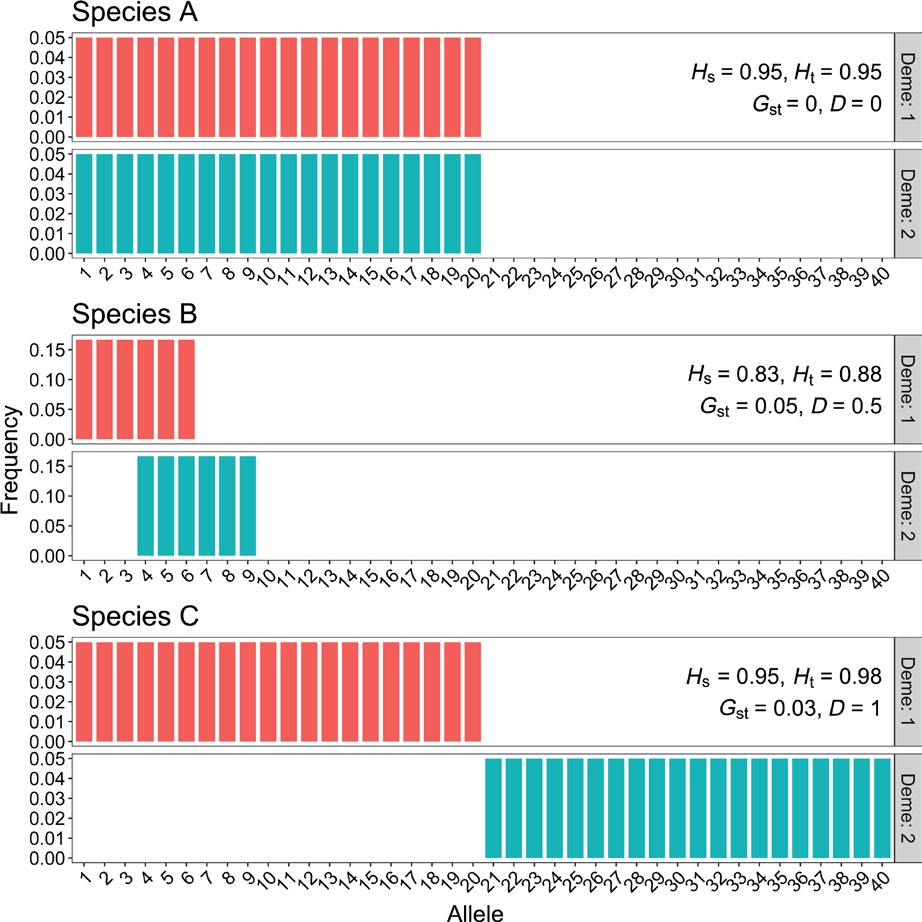
Three species with differing allelic differentiation. *G*_ST_ is close to zero in all three species populations but *D* ranges from zero (top) to 1.00 (bottom). All alleles occur at equal frequencies in each deme

At the other extreme, *G*_ST_ approaches zero when the demes have high heterozygosity or are identical in allele composition and frequencies (unless all demes are fixed for the same allele, in which case *G*_ST_ is undefined). When within‐deme heterozygosity is high, it does not matter much whether all the demes are identical or all the demes share no alleles; *G*_ST_ approaches zero in either case. In contrast, *D* and its relatives will approach zero if and only if all demes are identical and will be unity if and only if no demes share any alleles. In contrast to *G*_ST_, *D* is never undefined. Again, we emphasize that the population structures that minimize *G*_ST_ are not the same as the population structures that minimize *D*. For instance, consider three species, each with two demes (Figure 3). Species A, whose two demes share the same 20 alleles, each at the same low frequency, would have both *G*_ST_ and *D* equal to zero. Species B, whose two demes each share half of their 6 alleles with each other, would have *D *=* *0.5 and *G*_ST_ = 0.05. Species C, with 20 equally common alleles in each deme but with no shared alleles, has a *G*_ST_ of 0.03 (lower than that of species B) and *D *=* *1.0. A conservation manager who interpreted *G*_ST_ as a measure of allelic differentiation would probably have chosen not to preserve both demes of Species C, even though this is the species that would have most benefited by preserving both demes. Such a manager might have chosen instead to preserve both demes of Species B if resources were available, as this species has the largest value of *G*_ST_. The counterintuitive behavior of *G*_ST_ in this example is easily understood when one recognizes that *G*_ST_ measures nearness to fixation.

**Figure 4:**
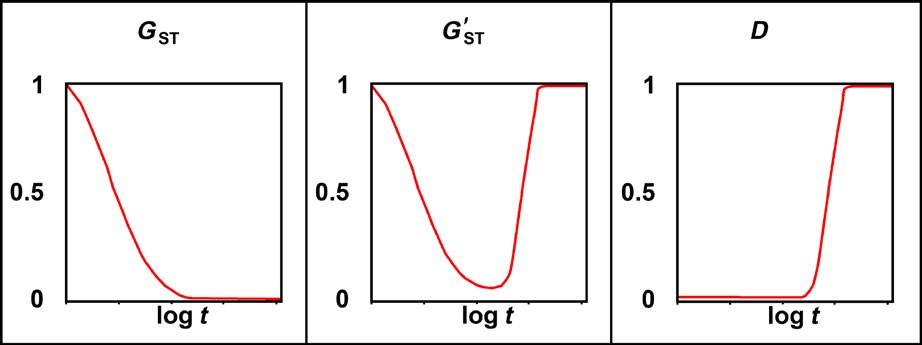
*G*_ST_, Hedrick's $G_{\mathrm{ST}}^{\prime }$ (Hedrick, 2005), and *D* (Jost, 2008), for 100 demes of 10,000 individuals, evolving under the finite island model with zero migration and a mutation rate of 0.001. Initially, 99 demes are fixed for the same allele and one deme is fixed for a different allele. The infinite‐allele model is used. Results are broadly similar to other models

It may also be helpful to consider a dynamic example. We choose an extreme case to illustrate the problem of interpreting *G*_ST_ in an evolutionary or conservation context; a more realistic case would show a similar but less pronounced pattern. Suppose an initially continuous population experiences a severe bottleneck event that also splits it into 100 very small demes with zero migration between them. Then the demes quickly recover so that at *t* = 0 they each contain 10,000 individuals, still with zero migration between them. Suppose that, due to founder effects, 99 of the demes are fixed for a single allele at a neutral locus, while one is fixed for a different allele (this odd deme is needed to keep *G*_ST_ from being undefined). Suppose the neutral locus has a high mutation rate of 0.001 substitutions per generation. Let this system evolve under Wright's finite island model with infinite alleles. Eventually all demes will contain only private alleles, and each deme will have high diversity. Figure 4 shows the values of *G*_ST_, Hedrick's $G_{\mathrm{ST}}^{\prime }$, and *D* over the course of this evolution. As the demes begin to differentiate and diversify, *G*_ST_ does the opposite of what a measure of allelic differentiation should do: it drops monotonically from unity when almost all demes were identical to near zero when all demes consist entirely of private alleles. Hedrick's $G_{\mathrm{ST}}^{\prime }$, which is an ad hoc measure designed to make *G*_ST_ behave like a real measure of allelic differentiation when heterozygosity is high, fails to address the equally grave difficulty of interpreting *G*_ST_ as a measure of allelic differentiation when heterozygosity is low (Gregorius, 2010; Gregorius & Roberds, 1986). *D* behaves as one would expect of a measure of allelic differentiation, increasing monotonically from near zero to unity without being affected by the changing heterozygosity.

Depending on the value of within‐deme genetic diversity, *G*_ST_ and *D* may not attain their full ranges. For example, for purely mathematical reasons *G*_ST_ must always be less than 1−*H*_*S*_, even when the demes share no alleles. The demes could even belong to different species, with no interbreeding, and *G*_ST_ would still be unable to attain a value greater than 1−*H*_*S*_. At the purely descriptive level, this behavior is in fact correct, if we remember that *G*_ST_ quantifies nearness to fixation, and populations with low values of 1−*H*_*S*_ are far from fixation.

Similarly, when the number of alleles is smaller than the number of demes, *D* cannot attain its maximum value of 1.00. At the purely descriptive level, this is the correct behavior, if we recall that *D* quantifies the actual observed allelic differentiation. In this case some of the demes must share the same alleles and are therefore not completely differentiated from each other.

### 3.2 Bi‐allelic SNP case

An important low‐diversity case arises when SNPs are analyzed. In this case there are only two alleles. As in the infinite‐allele case, *G*_ST_ is close to unity whenever all demes are near fixation, even if almost all demes are fixed for the same allele. If there are ten demes, with nine demes fixed for one base and one deme fixed for a different base, *G*_ST_ equals unity whereas *D* equals 0.20 as in Species A in Figure 2. If half the demes were fixed for one base and half for the other, *G*_ST_ would still be 1.00 as all demes are fixed. In this case, allelic differentiation would be at the maximum possible for two alleles, and *D* would be 0.5 (as in Species B in Figure 2). When there are many demes and only two alleles, *D* cannot equal unity, but the lower maximum correctly indicates that some of the demes must be identical.

However, if there are only two demes, or if we are looking at pairwise comparisons between multiple demes, this ambiguity in *G*_ST_ does not arise. When there are only two alleles, *G*_ST_'s dependence on within‐group heterozygosity is unimportant. Therefore, for the common special case of just two demes and two alleles, both *G*_ST_ and *D* give similar answers. The two families of measures are in fact equal when both demes are fixed for different alleles (*G*_ST_ = *D *=* *1.00). They are also equal when allele frequencies in both demes are identical (*G*_ST_ = *D *=* *0), except in the case when both demes are fixed for the same allele. (In that case *G*_ST_ is undefined because of division by zero.) *G*_ST_ and *D* are also equal when one deme contains equal proportions of each of the two alleles and the other deme is fixed for one of the alleles (*G*_ST_ = *D *=* *0.3333). They differ in some other cases, but broadly speaking, members of both families provide insight into differentiation for pairwise analyses of SNP data, although *D* has the advantage of never being undefined.

If the goal is description of genetic differentiation at multiple hierarchical levels, measures of allelic differentiation based on entropy rather than heterozygosity have some advantages over either *D* or *G*_ST_, because of their ease of decomposition across hierarchical levels. See Gaggiotti et al. (2018).

## 4 CONNECTION BETWEEN ALLELIC DIFFERENTIATION AND GENETIC DIVERSITY

Geneticists commonly use expected heterozygosity as a measure of diversity, and sometimes refer to it as “genetic diversity” or “gene diversity.” If the mean within‐deme *H*_*S*_ is close to the total population's *H*_*T*_ (in other words, if *G*_ST_ is close to zero), some conservation geneticists might conclude that most of the diversity is within demes, and protecting only one or a few demes could save most of the population's genetic diversity. While this conclusion seems simple and straightforward, every step leading to it is mathematically and biologically incorrect. As we have shown in the preceding sections, whenever within‐deme heterozygosity *H*_*S*_ is high (close to 1.00), its value is necessarily close to the total population heterozygosity *H*_*T*_, which cannot exceed unity, even if the demes share no alleles at all.

One of the problems with the classical reasoning is that heterozygosity lacks an essential property for a biological diversity measure that will be subject to ratio comparisons. If one wants to infer genetic similarity from a ratio comparison of within‐group diversity to total diversity, the diversity measure must be linear with respect to pooling of equally large, equally diverse, completely distinct groups. Heterozygosity lacks this property. Converting either *H*_*T*_ or *H*_*S*_ to Kimura and Crow's effective number of alleles (Kimura & Crow, 1964), through the formula 1/(1−H), creates a true diversity measure that does have this property.

Another problem with the classical reasoning is the assumption that *H*_*T*_ −* H*_*S*_ is the between‐group component of diversity. Some measures of compositional complexity, like Shannon entropy, are additive, but heterozygosity is subadditive. The correct way to partition heterozygosity into independent within‐ and between‐group components *H*_*S*_ and *H*_ST_ is by the formula *H*_*T*_ = *H*_*S*_ + *H*_ST_ −* H*_*S*_ *H*_ST_, assuming that each deme is given equal statistical weight (Jost, 2007). This is the formula that leads to *D*, which is just the normalized value of this *H*_ST_ (Equation (3)). The partitioning of effective number of alleles, in contrast, is multiplicative; it too leads to *D* as a measure of differentiation (Jost, 2008). Thus, if our goal is to conserve genetic diversity, we should measure genetic diversity by effective number of alleles and partition it correctly; *D* is the between‐group component of the correct partitioning of either heterozygosity or effective number of alleles, normalized onto the unit interval. A similar conversion to effective number of alleles makes Shannon entropy a legitimate diversity measure, which can also be partitioned multiplicatively; see Gaggiotti et al. (2018).

However, there are still unsolved problems with the conservation goal of maximizing genetic diversity. These are beyond the scope of the present article to solve, but one major issue concerns which “unit” of genetic diversity to try to preserve: SNPs, functional alleles, unique pairs of alleles (for diploid organisms), or genotypes (combinations of genes). Every additional allele that is preserved could potentially occur in combination with other alleles at that locus and at other loci, and each new combination could have a downstream phenotypic and fitness consequence. Thus, there is an argument for preserving genotypes, and in fact, genotypes are the focus of preservation in many crop conservation collections where a specific phenotype must be maintained. However, the number of possible combinations of alleles across multiple loci quickly becomes huge, and thus preserving every combination is not possible. Questions concerning which genotypic combinations to preserve, or what kind of diversity should be maximized, remain an unsolved problem for future work.

## 5 COMPARING *G*_ST_ AND *D* IN A SIMPLE CONSERVATION GENETICS SCENARIO

Clearly, *D* and *G*_ST_ do not provide the same information about populations. However, they can be used in a complementary manner—comparing them can provide useful information for conservation geneticists. We encourage researchers to calculate both and interpret them in the light of their different insight into fixation and allelic differentiation.

When considering a conservation strategy for a subdivided population, a manager will want to know how different are the demes. Is it worth investing scarce resources in preserving multiple demes, or is it enough to protect just one? We here only examine the genetic aspects of this question, recognizing that there are often other good reasons for wanting to conserve multiple demes. One way to answer this question is to measure directly the genetic diversity and differentiation of this population at coding loci that are expected to be important to the species’ survival, such as MHC loci that confer resistance to new diseases. Although one locus is typically insufficient for driving conservation action, adaptive loci are often important in the decision‐making process (see Flanagan, Forester, Latch, Aitken, & Hoban, 2018). To understand the mathematical issues involved, consider an artificial example of a population with two equally large demes, each with many low‐frequency alleles, and with almost no alleles shared between the demes (the hypothetical allele frequencies are given in Table S1).

The standard analysis might use heterozygosity as the measure of diversity, and would compare the total heterozygosity (*H*_*T*_), 0.97, to the within‐group heterozygosity (*H*_*S*_) 0.95. As 98% of the heterozygosity is within‐group (according to the usual, incomplete additive partitioning of heterozygosity), this analysis suggests the amount of differentiation among demes is relatively low. This thinking is reinforced by the value of *G*_ST_, which is only 0.02. Yet both demes consist almost entirely of private alleles. As shown earlier, *G*_ST_ is (correctly) indicating that demes are far from fixation, which clearly has nothing to do with allelic differentiation.

The mathematically correct analysis of allelic differentiation would convert heterozygosity to effective number of alleles 1/(1−*H*) and use that as the measure of diversity. The total diversity is 38.8 and the within‐group diversity is 20.4. The total diversity of the two pooled demes is almost twice the mean diversity of a single deme. As there are only two demes, this indicates that there are almost no alleles in common between them. The differentiation measure *D* takes this into account and has a value of 0.95, correctly indicating the high degree of allelic differentiation.

When there are more than two demes, pairwise *D* would correctly identify the demes whose allele frequencies differed most. Pairwise *G*_ST_ would identify demes nearest to fixation, rather than demes with different allele frequencies, as in the two‐deme case, which might be considered an erroneous conservation decision. Some authors (Strand et al., 2012) have noted that in pairwise application, *D* and *G*_ST_ may be strongly correlated, and so they choose to utilize just one of these measures. Yet the absolute magnitude of the differentiation or fixation is usually what matters to conservation managers. It is important to choose the measure whose magnitude reflects the effect of interest, and then interpret the actual magnitudes, not just the ranking or the statistical significance (Jost, 2009). It is also important to note that *H*_*T*_ is likely to vary among pairwise comparisons of demes, thus pairwise estimates of *G*_ST_ may not be truly comparable. On the other hand, the same value of *D* in pairwise comparisons (or between different species for that matter) can be interpreted to describe the same relative degree of allelic differentiation, making *D* a useful metric for comparisons both across and within species.

The allelic differentiation measures based on allele number (*K*_ST_, Equation (8)) or on entropy (*E*_ST_, Equation (7)) give additional useful information. For the data in Table S1, the differentiation based on allele number (*K*_ST_) is 0.77, still indicating high differentiation, but less than that indicated by the heterozygosity‐based differentiation *D* (*D *=* *0.95). This shows that on average, 77% of each deme's alleles are unique to that deme. The entropy‐based differentiation *E*_ST_ is 0.90, slightly less than the *D* of 0.95. Taken together, the values of these three differentiation measures show that the most frequent alleles in each deme, which are the ones most emphasized by *D*, show more differentiation than the average alleles, which are the ones emphasized by entropy, and both those categories show more differentiation than the class of all alleles without regard to frequency (*K*_ST_), showing that the shared alleles are found at very low frequencies in the population.

The differentiation based on allele number and differentiation based on *D* can often give highly divergent answers. Consider the set of allele frequencies for two demes given in Table 1. Here the value of *D* is 0.99 while the value of *K*_ST_ is zero. The high value of *D* indicates that the most common alleles are not shared, while the zero value of *K*_ST_ indicates that all alleles are in fact shared (none are unique to that deme), even though their frequencies are very different in each deme. Conservation managers should consider both results when forming conservation strategies.

**Table 1:** Allele frequencies for two demes

Allele	Deme 1	Deme 2
1	0.95	0.005
2	0.005	0.005
3	0.005	0.005
4	0.005	0.005
5	0.005	0.005
6	0.005	0.005
7	0.005	0.005
8	0.005	0.005
9	0.005	0.005
10	0.005	0.005
11	0.005	0.95

The two demes share all 11 alleles.

## 6 CONCLUSIONS

The two main families of measures of population structure yield different information about species of conservation concern. We urge researchers to interpret *G*_ST_ and other fixation indices as measures of nearness to fixation (as suggested by Wright himself) and *D* and similar metrics as measures of allelic differentiation. As noted earlier in the text, pairwise comparisons using bi‐allelic SNP markers result in similar values for *D* and *G*_ST_, so the distinction between the two families of measures is not as critical to the interpretation of the results. However, in all other cases, such as comparing SNPs across multiple populations or in comparisons at more polymorphic loci, *D* and *G*_ST_ provide different information. Calculations should be made for both neutral and putatively adaptive markers (those suspected or known to influence fitness), providing a more complete picture of genetic structure (Funk, McKay, Hohenlohe, & Allendorf, 2012). Sensible conservation decisions can be based directly on measurements at loci of conservation interest (e.g., MHC loci) without the need for equilibrium assumptions or other complex inferences. Decisions should be based on analysis of the actual magnitudes of these measures (and their confidence intervals), not on their statistical significance relative to an always‐false null model (Gilner, Morgan, Leech, & Harmon, 2001; Jost, 2009).

The absolute number of migrants per generation controls nearness to fixation (as long as mutation rate is low), not allelic differentiation. Note also that the effect of mutation on *D* increases as the number of demes increases, while this is not the case for *G*_ST_. This further illustrates the fact that the two measures provide very different but complementary types of information and should be used simultaneously in conservation genetics. Contrary to frequent misinterpretations, *D* is not intended as an estimator of *F*_ST_, and always tracks the actual present‐day heterozygosity‐weighted allelic differentiation between demes.

In most cases for conservation of threatened species, allelic differentiation measures will provide the information that is most relevant for choosing which subpopulations to protect. *D* is the allelic differentiation measure that has the simplest connection to genetic models and is the easiest measure to estimate reliably from small samples, but entropy differentiation (*E*_ST_, Equation (7)) has the most robust monotonicity and partitioning properties (Gaggiotti et al., 2018), and the differentiation measure based on allele number (*K*_ST_, Equation (8)) provides additional useful information.

Conservation decisions are complex, and measures of population structure by themselves cannot tell the whole story. Diversity, as measured by effective number of alleles (Hill numbers), provides additional important insights. Both diversity and differentiation can also be generalized to incorporate information about the degree of genetic differentiation between alleles (Chao, Chiu, & Jost, 2010; Gaggiotti et al., 2018). Combining input from multiple measures will produce the most effective conservation decisions.
