# Supplementary material for: Differentiation measures for conservation genetics
Source: Evol Appl. 2018 Jan 29;11(7):1139–48. doi: 10.1111/eva.12590 (PMC6050183; doi:10.1111/eva.12590)
Supplement: Supplementary file 1 [file EVA-11-1139-s001.docx]

**Supplemental Materials**

We generated populations of simulated genetic data for combinations (scenarios) of demographic parameters using the coalescent simulator *fastsimcoal* (Excoffier and Foll 2011). We conducted simulations under two sets of parameter spaces:

For the first simulation, we drew random samples from bins of D and G_ST_ representing low (0:0.1), medium (0.45:0.55), and high (0.9:1) values. In order to ensure even sampling within the bins, scenarios were selected using the following procedure:

1. Select 10_­_^8^ combinations of values from the following distributions:

N_e_ (effective population size) ~ 10^Uniform(log10(20), log10(500))^

d (number of demes) ~ 10^Uniform(log10(2), log10(500))^

µ (mutation rate) ~ 10^Uniform(-9, -1)^

D ~ (Uniform(0, 0.1) , Uniform(0.45, 0.55), Uniform(0.9, 1))^1^

G_ST_ ~ (Uniform(0, 0.1) , Uniform(0.45, 0.55), Uniform(0.9, 1))^1^

2. For each set of parameters from Step 1, calculate migration rates given the randomly selected values of D and G_ST_:

$$m_{D}=\left( \frac{1}{D}-1 \right)\mu(d-1)$$

$$m_{Gst}=\frac{\frac{1}{Gst}-1-4Ne\mu\left( \frac{d}{d-1} \right)}{4Ne\left( \frac{d}{d-1} \right)^{2}}$$

3. Calculate an “average migration rate” (m) for each scenario as the mean of m_D_ and m_GST_.

4. Using this value of m, calculate new values of expected D and G_ST_ (E(D) and E(G_ST_)) for each scenario following Equations 2 and 4 in the text.

5. Discard scenarios with E(D) or E(G_ST_) that are not within the ranges of 0:0.1, 0.45:0.55, or 0.9:1.

6. Select a random 1000 scenarios from each of the 9 bins of E(D) and E(G­_ST_).

The second simulation was based on combinations of m/μ(d-1) = 0.05, 1, 20, and Nm = 0.1, 1, 10. From the same distributions as above, we drew 1000_­_ random samples of N_e_ and d for each of the nine combinations. For each scenario, migration rate (m) was calculated as Nm / N_e_, and µ was then calculated from the randomly selected d, m, and the value of m/μ(d-1) in each combination.

For coalescent simulations of the 9000 scenarios in each set of simulations, an island migration matrix was used, and all demes were set to diverge from a common ancestor 10^5^ generations in the past. We generated genotypes for a single diploid locus mutating under the infinite alleles model for all individuals in the population. All computations were conducted using the R package *strataG* (Archer et al. 2016).

^1^For each scenario, the initial values of D and G_ST_ were selected by first choosing a random value from each of the three bins. One of these values was then randomly selected.

Archer, F. I., Adams, P. E. and Schneiders, B. B. (2016) strataG: An R package for manipulating, summarizing and analysing population genetic data. Mol Ecol Resour. doi:10.1111/1755-0998.12559

Excoffier, L. and Foll, M (2011) fastsimcoal: a continuous-time coalescent simulator of genomic diversity under arbitrarily complex evolutionary scenarios Bioinformatics 27: 1332-1334.

Table S1. Hypothetical allele frequencies for two equally large demes used in the simple conservation genetics scenario. In this scenario, demes are far from fixation (G_ST_ = 0.02). Allelic differentiation is high; the few alleles that are shared between demes are not common alleles (D = 0.95) or average alleles (E_ST_ = 0.90) but are low-frequency alleles (K_ST_ = 0.77).

|  | Deme 1 | Deme 2 |
| --- | --- | --- |
| Allele 1 | 0.05 | 0.00 |
| Allele 2 | 0.05 | 0.00 |
| Allele 3 | 0.05 | 0.00 |
| Allele 4 | 0.05 | 0.00 |
| Allele 5 | 0.05 | 0.00 |
| Allele 6 | 0.05 | 0.00 |
| Allele 7 | 0.05 | 0.00 |
| Allele 8 | 0.05 | 0.00 |
| Allele 9 | 0.05 | 0.00 |
| Allele 10 | 0.05 | 0.00 |
| Allele 11 | 0.05 | 0.00 |
| Allele 12 | 0.05 | 0.00 |
| Allele 13 | 0.05 | 0.00 |
| Allele 14 | 0.05 | 0.00 |
| Allele 15 | 0.05 | 0.00 |
| Allele 16 | 0.05 | 0.01 |
| Allele 17 | 0.05 | 0.01 |
| Allele 18 | 0.05 | 0.01 |
| Allele 19 | 0.05 | 0.01 |
| Allele 20 | 0.05 | 0.01 |
| Allele 21 | 0.00 | 0.05 |
| Allele 22 | 0.00 | 0.05 |
| Allele 23 | 0.00 | 0.05 |
| Allele 24 | 0.00 | 0.05 |
| Allele 25 | 0.00 | 0.05 |
| Allele 26 | 0.00 | 0.05 |
| Allele 27 | 0.00 | 0.05 |
| Allele 28 | 0.00 | 0.05 |
| Allele 29 | 0.00 | 0.05 |
| Allele 30 | 0.00 | 0.05 |
| Allele 31 | 0.00 | 0.05 |
| Allele 32 | 0.00 | 0.05 |
| Allele 33 | 0.00 | 0.05 |
| Allele 34 | 0.00 | 0.05 |
| Allele 35 | 0.00 | 0.05 |
| Allele 36 | 0.00 | 0.05 |
| Allele 37 | 0.00 | 0.05 |
| Allele 38 | 0.00 | 0.05 |
| Allele 39 | 0.00 | 0.00 |
| Allele 40 | 0.00 | 0.05 |
